# Static compliance and driving pressure are associated with ICU mortality in intubated COVID-19 ARDS

**DOI:** 10.1186/s13054-021-03667-6

**Published:** 2021-07-28

**Authors:** Annalisa Boscolo, Nicolò Sella, Giulia Lorenzoni, Tommaso Pettenuzzo, Laura Pasin, Chiara Pretto, Martina Tocco, Enrico Tamburini, Alessandro De Cassai, Paolo Rosi, Enrico Polati, Katia Donadello, Leonardo Gottin, Silvia De Rosa, Fabio Baratto, Fabio Toffoletto, V. Marco Ranieri, Dario Gregori, Paolo Navalesi, COVID-19 VENETO ICU Network, Ilaria Valeri, Giulio Andreatta, Leonardo Gandolfi, Alessandra Gadaldi, Nicolò Brumana, Edoardo Forin, Christelle Correale, Elisa Pesenti, Davide Fregolent, Pier Francesco Pirelli, Davide Marchesin, Matteo Perona, Nicola Franchetti, Michele Della Paolera, Caterina Simoni, Tatiana Falcioni, Alessandra Tresin, Chiara Schiavolin, Aldo Schiavi, Sonila Vathi, Daria Sartori, Alice Sorgato, Elisa Pistollato, Federico Linassi, Eugenio Serra, Demetrio Pittarello, Ivo Tiberio, Ottavia Bond, Elisa Michieletto, Luisa Muraro, Arianna Peralta, Paolo Persona, Enrico Petranzan, Francesco Zarantonello, Alessandro Graziano, Eleonora Piasentini, Lorenzo Bernardi, Roberto Pianon, Flavio Badii, Enrico Bosco, Moreno Agostini, Antonio Farnia, Mario Peta, Mauro Antonio Calò, Marco Meggiolaro, Francesco Lazzari, Ivan Martinello, Giorgio Fullin, Francesco Papaccio, Alfeo Bonato, Camilla Sgarabotto, Francesco Montacciani, Parnigotto Alessandra, Giuseppe Gagliardi, Gioconda Ferraro, Luigi Ongaro, Marco Baiocchi, Vinicio Danzi, Paolo Zanatta, Simonetta Marchiotto, Silvia Bassanini, Massimo Zamperini, Ivan Daroui

**Affiliations:** 1 Institute of Anaesthesia and Intensive Care Unit, Padua University Hospital, via V. Gallucci 13, 35125 Padua, Italy; 2 Department of Medicine (DIMED), Padua University School of Medicine, Padua, Italy; 3 Unit of Biostatistics, Epidemiology and Public Health, Department of Cardiac, Thoracic, Vascular Sciences and Public Health, Padua University School of Medicine, Padua, Italy; 4 Emergency Medical Services, Regional Department, AULSS 3, Venice, Italy; 5 Anesthesia and Intensive Care Unit B, Verona University Hospital, Verona, Italy; 6 Anesthesia and Critical Care Unit, San Bortolo Hospital, Vicenza, Italy; 7 Anesthesia and Intensive Care Unit, Ospedale Riuniti Padova Sud, Schiavonia, Italy; 8 Anesthesia and Intensive Care Unit, Ospedali di San Donà di Piave e Jesolo, San Donà di Piave, Italy; 9 Anesthesia and Intensive Care Medicine, Department of Medical and Surgical Science, Policlinico di Sant’Orsola, Alma Mater Studiorum-Università di Bologna, Bologna, Italy

**Keywords:** COVID-19, ARDS, Mechanical ventilation, Driving pressure, Respiratory system compliance

## Abstract

**Background:**

Pathophysiological features of coronavirus disease 2019-associated acute respiratory distress syndrome (COVID-19 ARDS) were indicated to be somewhat different from those described in nonCOVID-19 ARDS, because of relatively preserved compliance of the respiratory system despite marked hypoxemia. We aim ascertaining whether respiratory system static compliance (Crs), driving pressure (DP), and tidal volume normalized for ideal body weight (VT/kg IBW) at the 1st day of controlled mechanical ventilation are associated with intensive care unit (ICU) mortality in COVID-19 ARDS.

**Methods:**

Observational multicenter cohort study. All consecutive COVID-19 adult patients admitted to 25 ICUs belonging to the COVID-19 VENETO ICU network (February 28th–April 28th, 2020), who received controlled mechanical ventilation, were screened. Only patients fulfilling ARDS criteria and with complete records of Crs, DP and VT/kg IBW within the 1st day of controlled mechanical ventilation were included. Crs, DP and VT/kg IBW were collected in sedated, paralyzed and supine patients.

**Results:**

A total of 704 COVID-19 patients were screened and 241 enrolled. Seventy-one patients (29%) died in ICU. The logistic regression analysis showed that: (1) Crs was not linearly associated with ICU mortality (*p* value for nonlinearity = 0.01), with a greater risk of death for values < 48 ml/cmH_2_O; (2) the association between DP and ICU mortality was linear (*p* value for nonlinearity = 0.68), and increasing DP from 10 to 14 cmH_2_O caused significant higher odds of in-ICU death (OR 1.45, 95% CI 1.06–1.99); (3) VT/kg IBW was not associated with a significant increase of the risk of death (OR 0.92, 95% CI 0.55–1.52). Multivariable analysis confirmed these findings.

**Conclusions:**

Crs < 48 ml/cmH_2_O was associated with ICU mortality, while DP was linearly associated with mortality. DP should be kept as low as possible, even in the case of relatively preserved Crs, irrespective of VT/kg IBW, to reduce the risk of death.

**Supplementary Information:**

The online version contains supplementary material available at 10.1186/s13054-021-03667-6.

## Background

Pathophysiological features of coronavirus disease 2019 (COVID-19)-associated acute respiratory distress syndrome (COVID-19 ARDS) were indicated to be somewhat different from those described in nonCOVID-19 ARDS, because of relatively preserved compliance of the respiratory system despite marked hypoxemia [1, 2]. These preliminary observations led some authors to question the efficacy of lung protective ventilation in patients with COVID-19 ARDS and suggested that a less tight limitation of volume could be allowed while delivering mechanical ventilation to these patients [2]. Other authors, however, proposed that protective ventilation limiting volume and pressure should be applied also in COVID-19 ARDS [3]. While strong evidence showed that lung protective ventilation improves survival in nonCOVID-19 ARDS patients [4–6], data are still lacking on COVID-19 ARDS patients.

In order to assess whether lung protective ventilation affects intensive care unit (ICU) mortality also in patients with COVID-19 ARDS, we set up a study to test the hypothesis that static compliance of the respiratory system (Crs), driving pressure (DP), and tidal volume normalized for ideal body weight (VT/kg IBW) are associated with ICU mortality.

## Methods

This multicenter observational study was coordinated by Padua University Hospital (Italy), and followed the “Strengthening the Reporting of Observational Studies in Epidemiology” statement guidelines for observational cohort studies (Additional file 1) [7]. The study was approved by the Institutional Ethical Committee of each participating center (coordinator center approval reference number 4853AO20, while the full list of all approval reference numbers is available in Additional file 2) and informed consent was obtained according to the national regulation. Data were collected by the COVID-19 VENETO ICU Network, including 25 ICUs [8], and inserted into a pre-designed online data acquisition system (www.covid19veneto.it). Patients’ privacy was protected by assigning a de-identified patient code. Guidelines for management of mechanically ventilated patients had been provided to all ICUs of the regional network (from March 2nd, 2020) [8].

We included all consecutive adult patients with confirmed SARS-CoV-2 infection and fulfilling ARDS criteria [1], admitted between February 28th and April 28th, 2020, who received controlled mechanical ventilation (CMV), and had Crs, DP and VT/kg IBW measured in supine position, after sedation and paralysis, within the first 24 h of CMV. We excluded the patients with incomplete records, and those who had been prone positioned prior to data collection. COVID-19 diagnosis was made according to the WHO interim guidance (http://www.who.int/docs/default-source/coronaviruse/clinical-management-of-novel-cov.pdf).

The following variables were collected: (1) demographic data (age, gender, body mass index); (2) Charlson comorbidity index not adjusted for age; (3) sequential organ failure assessment (SOFA) score at ICU admission; (4) gas exchange within the first 24 h of CMV [pH, arterial partial pressure of oxygen (PaO_2_), arterial partial pressure of carbon dioxide (PaCO_2_)]; (5) ventilator settings [VT/kg IBW, respiratory rate, positive end-expiratory pressure (PEEP), fraction of inspired oxygen (FiO_2_)]; (6) plateau pressure, DP, Crs; and (7) ICU mortality.

Ventilator settings, plateau pressure, DP and Crs were collected within the first 24 h after endotracheal intubation in supine position on the basis of the senior attending physician’s assessment. The measurements were performed in sedated and paralyzed patients, with no spontaneous breathing activity. Patients were ventilated in volume-controlled mode. Plateau pressure and total PEEP were measured at zero flow point during end-inspiratory and end-expiratory pauses [5, 6]. DP was calculated as the difference between plateau pressure and total PEEP, while Crs was calculated as VT/DP [5, 6]. In the case of multiple determinations, the attending clinician in charge for the patients identified the most representative set of variables to be included in the analysis [14].

Prior to data analysis, two independent investigators and a statistician screened the database for errors against standardized ranges and contacted local investigators for any queries. Validated data were then entered into the final database. Patients transferred from one ICU to another were considered as a single case, while for those readmitted to ICU after discharge, only data from the first admission were considered.

### Statistical analysis

Categorical data are presented as absolute numbers (n) and percentages (%). For continuous data, normality was tested by Skewness and Kurtosis tests. Means and standard deviations are used for normally distributed variables, while medians and interquartile ranges are used for non-normally distributed variables. No imputation for missing data has been planned. Logistic regression models have been estimated to assess the association between ICU mortality and Crs, DP, VT/kg IBW, total PEEP and plateau pressure. If the association was nonlinear, restricted cubic splines have been used to estimate the models and the change-point has been identified.

Additionally, multivariable logistic regression models have been used to evaluate the association between Crs and DP and ICU mortality after adjusting for relevant confounders. In each model, the independent predictors have been identified through a stepwise regression approach. This approach combines forward and backward selection methods in an iterative procedure (with a significance level of 0.05 both for entry and retention) to select predictors in the final multivariable model [9]. Independent variables used in the stepwise approach were age, gender, body mass index, SOFA score at ICU admission, Charlson comorbidity index, pH, PaO_2_/FiO_2_, PaCO_2_, ventilator settings (VT/kg IBW, respiratory rate, total PEEP) and plateau pressure at the first day of CMV. Additionally, collinearity has been defined for GVIF^(1/(2×Df))^ value greater than 2 [9, 10]. Variables with multicollinearity have been removed from the model (Additional file 3). Two different multivariable logistic regression models, one for Crs (model 1) and one for DP (model 2), were generated.

All statistical tests were 2-tailed, and statistical significance was defined as *p* < 0.05. All analyses have been conducted using R version 4.0.3 (R foundation for Statistical Computing, Vienna, Austria).

## Results

**Fig. 1:**
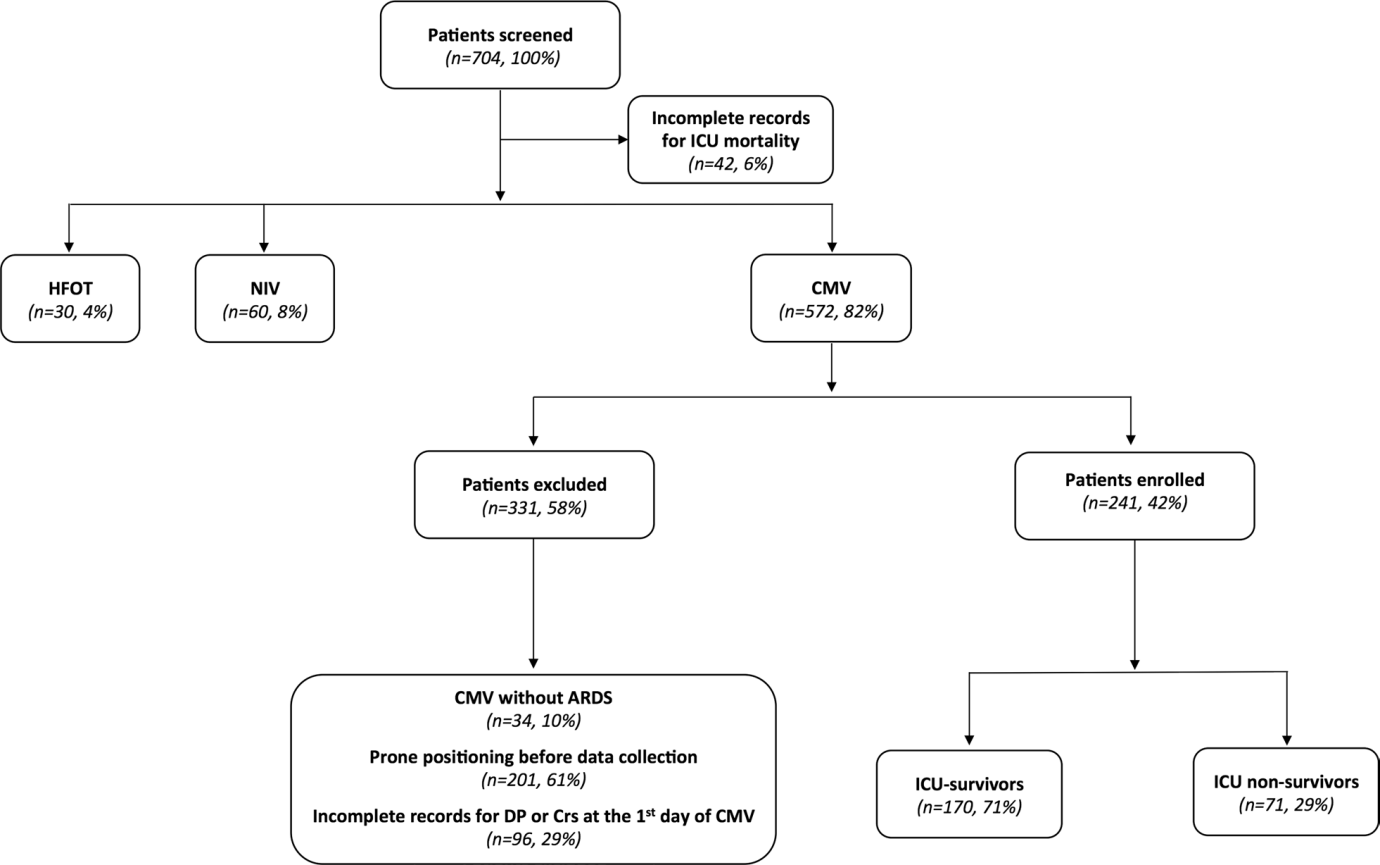
Flow chart of enrolled patients. *ICU* intensive care unit, *HFOT* high flow oxygen therapy, *NIV* non-invasive ventilation, *CMV* controlled mechanical ventilation, *ARDS* acute respiratory distress syndrome, *DP* driving pressure, *Crs* static compliance of the respiratory system

During the study period, a total of 704 consecutive COVID-19 ARDS patients from 25 ICUs were included in the database. After excluding 463 patients, who did not receive CMV or did not fulfill ARDS criteria [1] or did not have complete records in supine position, 241 patients from 21 ICUs were deemed eligible for data analysis (Fig. 1).

Demographic and clinical characteristics of the study population are listed in Table 1.

**Table 1:** Clinical characteristics, respiratory parameters and outcomes of the study population

	Overall population *n* = 241
*Clinical characteristics*	
Age (years)	66 [58–73]
Gender (male)	189 (78%)
SOFA score at ICU admission	5 [4–8]
BMI (kg/m^2^)	27 [25–30]
Charlson comorbidity index	1 [1, 2]
Onset of symptoms (days)	7 [3–9]
Hospital LOS before ICU (days)	2 [1–5]
Hospital LOS before CMV (days)	2 [1–5]
*Gas exchange, at first day of CMV*	
pH	7.41 [7.36–7.46]
PaO_2_/FiO_2_ (mmHg)	142 [102–216]
PaCO_2_ (mmHg)	44 [38–52]
*Ventilator settings and variables, at first day of CMV*	
Tidal volume (ml/kg of ideal body weight)	7.8 [6.9–8.8]
Set respiratory rates (breaths/min)	16 [14–20]
Total PEEP (cmH_2_O)	12 [10–13]
FiO_2_ (%)	60 [50–70]
Plateau pressure (cmH_2_O)	23 [20–26]
Driving pressure (cmH_2_O)	11 [9–13]
Static compliance of the respiratory system (ml/cmH_2_O)	48 [39–60]
*Respiratory treatments before CMV*	
NIV failure	110 (46%)
HFOT failure	27 (11%)
None	104 (43%)
*Adjunctive therapies during ICU stay*	
Prone position	202 (84%)
Neuromuscular blockers > 24 h	212 (88%)
V–V ECMO	5 (2%)
V–A ECMO	1 (0.4%)
*Outcomes*	
ICU mortality	71 (29%)
60-day mortality	78 (32%)
Length of CMV (days)	12 [7–16]
ICU LOS (days)	15 [9–25]
Hospital LOS (days)	30 [18–41]

Data are expressed as median and interquartile range [IQR] or number (%)

*SOFA* sequential organ failure assessment, *ICU* intensive care unit, *BMI* body mass index, *LOS* length of stay, *CMV* controlled mechanical ventilation, *PaO*_*2*_ partial pressure of arterial oxygen, *PaO*_*2*_*/FiO*_*2*_ ratio between partial pressure of arterial oxygen and fraction of inspired oxygen, *PaCO*_*2*_ partial pressure of carbon dioxide, *PEEP* positive end-expiratory pressure, *FiO*_*2*_ fraction of inspired oxygen, *NIV* non-invasive ventilation, *HFOT* high flow oxygen therapy, *V–V* veno–venous, *V–A* veno–arterial, *ECMO* extracorporeal membrane oxygenation

On the first day of CMV, median Crs was 48 (39–60) ml/cmH_2_O, DP 11 (9–13) cmH_2_O and VT/kg IBW 7.8 [6.9–8.8) ml/kg. Seventy-one patients (29%) died in ICU.

**Fig. 2:**
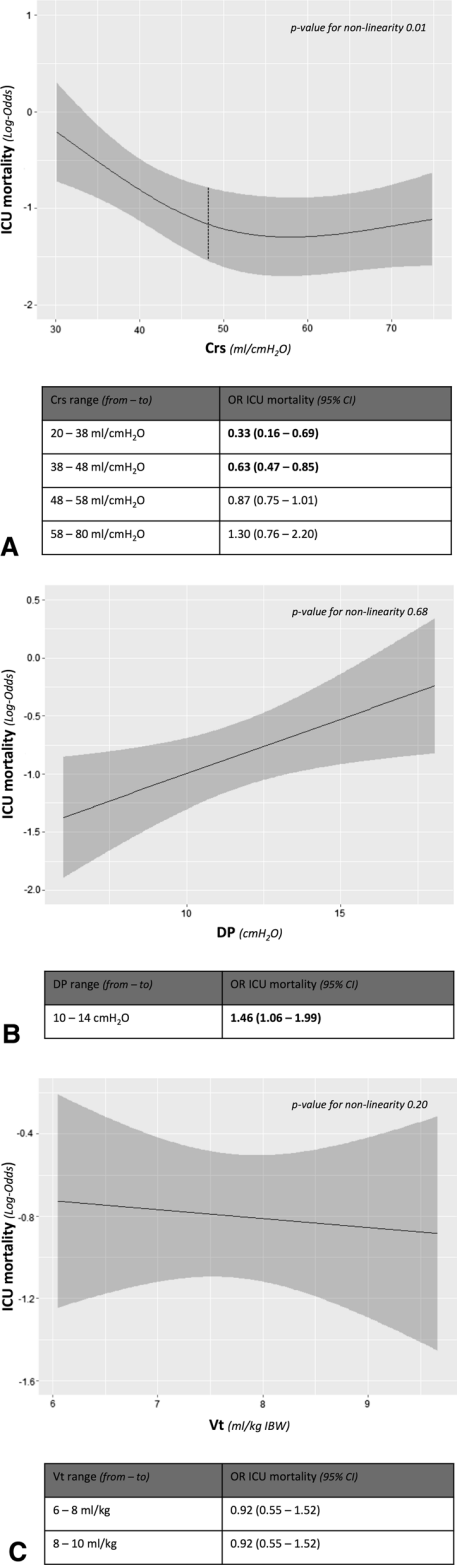
Solid lines indicate the log-odds of ICU mortality, while grey areas 95% confidence interval. **A** Association between static compliance of the respiratory system and intensive care unit mortality. The *p* value for nonlinearity was 0.01. The nonlinear effect of static compliance of the respiratory system on the intensive care unit mortality risk was modelled using restricted cubic splines. The odds ratio is presented for the interquartile ranges of the study population. **B** Association between driving pressure and intensive care unit mortality. Because *p* value for nonlinearity was 0.68, nonlinearity was not implemented in the model. The odds ratio is presented for clinically relevant range of values, according to published data [5, 6]. **C** Association between tidal volume and intensive care unit mortality. Because *p* value for nonlinearity was 0.20, nonlinearity was not implemented in the model. The odds ratio is presented for clinically relevant range of values, according to literature data [2, 4]. *ICU* intensive care unit, *Crs* static compliance of the respiratory system, *DP* driving pressure, *Vt* tidal volume, *IBW* ideal body weight, *OR* odds ratio, *95% CI* 95% confidence interval

The logistic regression analysis revealed a nonlinear relationship between Crs and ICU mortality (*p* value for nonlinearity = 0.01), with a significantly greater risk of death for values below 48 ml/cmH_2_O (Fig. 2A).

Contrariwise, a linear relationship between DP and ICU mortality (*p* value for nonlinearity = 0.68) was observed. An increase of DP from 10 to 14 cmH_2_O was associated with a 45% increment of the risk of ICU mortality (OR 1.45, 95% CI 1.06–1.99) (Fig. 2B). In addition, a linear relationship was also confirmed between ICU mortality and both total PEEP and plateau pressure (*p* value for nonlinearity = 0.22 both) (Additional file 4A, B).

VT/kg IBW was not associated with ICU mortality. Increasing VT/kg IBW from 6 to 8 ml/kg or from 8 to 10 ml/kg did not increase the risk of death (OR 0.92, 95% CI 0.55–1.52, for both ranges) (Fig. 2C).

Multivariable analysis confirmed Crs and DP to be independent risk factors for ICU mortality (OR 0.98, 95% CI 0.96–1.00, *p* = 0.03 and OR 1.12, 95% CI 1.00–1.24, *p* = 0.04, respectively) (Table 2).

**Table 2:** Multivariable models on the association between ICU mortality and static compliance of respiratory system (model 1) and driving pressure (model 2) at the first day of invasive controlled mechanical ventilation

	OR (95% CI)	*p* value*
*Model 1*		
Age (years)	1.10 (1.05–1.15)	< 0.01
Static compliance of the respiratory system (per ml/cmH_2_O increase)	0.98 (0.96–1.00)	0.03
*Model 2*		
Age (years)	1.10 (1.05–1.15)	< 0.01
Driving pressure (per cmH_2_O increase)	1.12 (1.00–1.24)	0.04

Data are expressed as Odds ratio (OR) and 95% Confidence Interval (CI)

^*^Stepwise regression approach, combining forward and backward selection methods (with a significance level of 0.05 both for entry and retention), was applied to select predictors in the final multivariable model. Independent variables finally entered in the multivariable models were age, gender, body mass index, SOFA score at ICU admission, Charlson comorbidity index, PaO_2_/FiO_2_, PaCO_2_, VT/kg IBW and respiratory rate. Multicollinearity analysis is reported as Additional file 3

## Discussion

We found that in COVID-19 ARDS patients (1) Crs has a nonlinear relationship with ICU mortality, with a greater risk of death for values below 48 ml/cmH_2_O; (2) DP is associated with ICU mortality, which increases linearly with DP increment; (3) VT/kg IBW is not a significant risk factor of ICU mortality.

In nonCOVID-19 ARDS patients, Crs failed to predict clinical outcomes, despite being associated with the functional lung size and the severity of the syndrome [5, 11]. A secondary analysis of the LUNG SAFE study, however, showed that lower Crs values, recorded on the first day of ARDS, were independently associated with mortality, even though the Crs–mortality relationship lacked a clear transition point and no useful cutoff could be established [9].

Wide ranges of Crs have been reported in COVID-19 ARDS patients [2, 12, 13], but the relationship between Crs and mortality remains uncertain [14, 15]. Grasselli et al. found that, among patients with COVID-19 ARDS, those who presented ‘low’ Crs within 24 h from ICU admission, associated with ‘high’ D-dimer concentration, had significantly greater 28-day mortality [14]. However, other studies were not able to confirm these results [13, 15].

We found Crs to be nonlinearly correlated with ICU mortality, with a significant association only for Crs values below 48 ml/cmH_2_O. This nonlinear relationship, which has already been reported in nonCOVID-19 ARDS patients [9], may depend on clinically relevant phenotypic heterogeneity [2, 3, 16, 17], and could explain the inconsistent results of previous investigations testing the association between Crs and mortality using a linear statistical approach [13–15].

DP has been extensively studied in nonCOVID-19 ARDS patients and was associated with poor clinical outcomes [5, 6]. Indeed, in a post hoc observational study including 3562 ARDS patients, DP proved to be the variable that best stratified 60-day mortality risk, irrespective of PEEP, plateau pressure and VT/kg IBW [5]. Furthermore, the LUNG SAFE study, which enrolled 2377 intubated ARDS patients, showed that DP > 14 cmH_2_O on the first day of CMV is a risk factor of poor hospital survival [6]. For patients without ARDS, the effect of DP on the clinical outcome is still controversial [18–20], though a meta-analysis including 2250 patients found that high DP during general anesthesia was associated with more postoperative pulmonary complications [21]. In COVID-19 ARDS patients, some cohort studies reported DP values quite close to those of nonCOVID-19 ARDS patients [12, 22], though the predictive role of DP on mortality remains unclear [13].

In our study FiO_2_ and PEEP were set according to the lower PEEP/FiO_2_ table [23]. It should be noted that PEEP settings were applied according to an official document released by the regional government and generated by consensus among clinicians of the ICU network, thus providing consistency and homogeneity to the data used for our analysis.

To the best of our knowledge, the present study is the first investigation finding a significant association between DP and mortality in COVID-19 ARDS patients receiving CMV. This linear relationship suggests that every effort should be made to reduce DP as much as possible, also in patients with relatively preserved Crs values. If, on the one hand, this is quite easy to accomplish when Crs is relatively preserved, on the other hand, when Crs is low, extracorporeal CO_2_ removal may be necessary to achieve this goal. Interestingly, the effect of DP on ICU mortality seems to be independent from VT/kg IBW, whose increment above the traditional cutoff value [4] did not affect patient outcome. Similar results have been recently reported in nonCOVID-19 ARDS patients by a post hoc secondary analysis of 5 randomized trials, including 1096 classical ARDS patients, which found that the benefit of lower tidal volumes (4–8 ml/kg IBW) on 60-day mortality was related to respiratory system elastance, suggesting that lung-protective ventilation strategies should primarily target driving pressure rather than tidal volume [24].

Our study has limitations. First, like the vast majority of the studies published during COVID-19 pandemic, it is an observational study, suffering the limits of this design. Second, we measured Crs and DP, which include the mechanical properties of the chest wall, in addition to those of the lung. Worth mentioning, however, a recent meta-analysis showed that more sophisticated respiratory parameters did not add important information on the risk of death in comparison to DP [25]. Third, our findings focus only on respiratory variables collected within the first day of CMV, while subsequent measurements during ICU stay were not considered. Although this approach has already been used by several published papers [6, 9, 14], it fails exploring the role of further clinical evolution after the first day. Moreover, the lead-time bias in the form of time period for which patients fulfilled ARDS criteria [1] or had ARDS before the assessment on the first day of CMV remains an unmeasured confounder [9]. Fourth, although regional guidelines proposed standardized ventilatory settings that were overall accepted [8], we cannot exclude for sure that some deviations from the indications occurred. Lastly, several patients were excluded for incomplete records, which depended on the overwhelming workload for ICU physicians during the COVID-19 pandemic making data recording problematic, especially in hospitals not included in the residency program.

## Conclusions

In COVID-19 ARDS patients receiving CMV, ICU mortality is associated with Crs < 48 ml/cmH_2_O and linearly associated with DP. Our results suggest that DP should be kept as low as possible, irrespective of VT/kg IBW, to reduce the risk of death.

## Supplementary Information

**Additional file 1.** STROBE Statement-Checklist.

**Additional file 2.** Ethics committee approval.

**Additional file 3.** Multicollinearity analysis.

**Additional file 4.** (**A**) Association between total positive end-expiratory pressure (PEEP) and intensive care unit (ICU) mortality. (**B**) Association between plateau pressure and intensive care unit (ICU) mortality.

## Data Availability

The data that support the findings of this study are available from the corresponding author, PN, upon request.

## Abbreviations

COVID-19: Coronavirus disease 2019
ARDS: Acute respiratory distress syndrome
ICU: Intensive care unit
Crs: Static compliance of the respiratory system
DP: Driving pressure
VT/kg IBW: Tidal volume normalized for ideal body weight
CMV: Controlled mechanical ventilation
SOFA: Sequential organ failure assessment score
PaO_2_: Arterial partial pressure of oxygen
PaCO_2_: Arterial partial pressure of carbon dioxide
PEEP: Positive end-expiratory pressure
FiO_2_: Fraction of inspired oxygen
