# Supplementary material for: Static compliance and driving pressure are associated with ICU mortality in intubated COVID-19 ARDS
Source: Crit Care. 2021 Jul 28;25:263. doi: 10.1186/s13054-021-03667-6 (PMC8317138; doi:10.1186/s13054-021-03667-6)
Supplement: Supplementary file 2 — Additional file 2. Ethics committee approval. [file 13054_2021_3667_MOESM2_ESM.pdf]

ADDITIONAL FILE 2

Title: Static compliance and driving pressure are associated with ICU mortality in intubated COVID-19 ARDS.

Authors: Annalisa Boscolo[1*] MD, Nicolò Sella[2*] MD, Giulia Lorenzoni[3] PhD, Tommaso Pettenuzzo[1] MD, Laura Pasin[1] MD, Chiara Pretto[2] MD, Martina Tocco[2] MD, Enrico Tamburini[2] MD, Alessandro De Cassai[1] MD, Paolo Rosi[4] MD, Enrico Polati[5] MD, Katia Donadello[5] MD, Leonardo Gottin[5] MD, Silvia De Rosa[6] MD, Fabio Baratto[7] MD, Fabio Toffoletto[8] MD, V. Marco Ranieri[9] MD, Dario Gregori[3] PhD, Paolo Navalesi[1,2] MD, FERS, for the COVID-19 VENETO ICU Network°.

**These authors equally contributed to this work.*

°Listed in the Acknowledgment section.

ADDITIONAL FILE 2. Ethics committee approval.

All the participating centres obtained ethics committee approval for the present research project (4853AO20 for Azienda Ospedale-Università di Padova; 2667CESC; 147A/CESC for AULSS 3 and 4; 2686-2687CESC; 76982CESC for AULSS 6; 2784CESC; 2781CESC; 2754CESC; 31/20ACESC; 801/CE for AULSS 2; 804/CE; 31/20B-C CESC for AULSS7). Informed consent was obtained for each patient according to the national regulation. In case the patient was not capable of giving informed consent at the time of enrolment, deferred consent was given. Consent to participate was waived for deceased patients, in accordance to rule 146/2019 of the Italian Privacy Authority. Local investigators were responsible for ensuring data integrity and validity.
