# Supplementary material for: Static compliance and driving pressure are associated with ICU mortality in intubated COVID-19 ARDS
Source: Crit Care. 2021 Jul 28;25:263. doi: 10.1186/s13054-021-03667-6 (PMC8317138; doi:10.1186/s13054-021-03667-6)
Supplement: Supplementary file 3 — Additional file 3. Multicollinearity analysis. [file 13054_2021_3667_MOESM3_ESM.pdf]

ADDITIONAL FILE 3

Title: Static compliance and driving pressure are associated with ICU mortality in intubated COVID-19 ARDS.

Authors: Annalisa Boscolo[1*] MD, Nicolò Sella[2*] MD, Giulia Lorenzoni[3] PhD, Tommaso Pettenuzzo[1] MD, Laura Pasin[1] MD, Chiara Pretto[2] MD, Martina Tocco[2] MD, Enrico Tamburini[2] MD, Alessandro De Cassai[1] MD, Paolo Rosi[4] MD, Enrico Polati[5] MD, Katia Donadello[5] MD, Leonardo Gottin[5] MD, Silvia De Rosa[6] MD, Fabio Baratto[7] MD, Fabio Toffoletto[8] MD, V. Marco Ranieri[9] MD, Dario Gregori[3] PhD, Paolo Navalesi[1,2] MD, FERS, for the COVID-19 VENETO ICU Network°.

**These authors equally contributed to this work.*

°Listed in the Acknowledgment section.

Additional file 3. Multicollinearity analysis.

	MODEL 1		MODEL 2
Age (years)	1.45	*Age (years)*	1.53
Gender (male)	1.33	*Gender (male)*	1.23
BMI (kg/m²)	1.62	*BMI (kg/m²)*	1.66
SOFA score at ICU admission	1.15	*SOFA score at ICU admission*	1.25
Charlson comorbidity index	1.19	*Charlson comorbidity index*	1.66
*pH**	-	*pH**	-
PaO_2/FiO_2 (mmHg)	1.27	*PaO_2/FiO_2 (mmHg)*	1.21
$PaCO_2$ (mmHg)	1.61	*$PaCO_2$ (mmHg)*	1.49
Tidal volume (ml/kg of ideal body weight)	1.67	*Tidal volume (ml/kg of ideal body weight)*	1.67
Set respiratory rates (breaths/min)	1.75	*Set respiratory rates (breaths/min)*	1.75
*Total PEEP (cmH_2O)**	-	*Total PEEP (cmH_2O)**	-
*Plateau pressure (cmH_2O)**	-	*Plateau pressure (cmH_2O)**	-
Static compliance of the respiratory system (ml/cmH_2O)	1.31	*Driving pressure (cmH_2O)*	1.21

The association between ICU mortality and static compliance of respiratory system was investigated by model 1, and the association between ICU mortality and driving pressure by model 2.

*: Variables excluded from model 1 and 2 due to multicollinearity, which was defined by $GVIF^{(1/(2 \times Df))}$ value greater than 2.

Abbreviations: BMI: body mass index; SOFA: sequential organ failure assessment; ICU: intensive care unit; PaO_2: partial pressure of arterial oxygen; PaO_2/FiO_2: ratio between partial pressure of arterial oxygen and fraction of inspired oxygen; $PaCO_2$: partial pressure of carbon dioxide; PEEP: positive end-expiratory pressure.
