# Supplementary material for: Static compliance and driving pressure are associated with ICU mortality in intubated COVID-19 ARDS
Source: Crit Care. 2021 Jul 28;25:263. doi: 10.1186/s13054-021-03667-6 (PMC8317138; doi:10.1186/s13054-021-03667-6)
Supplement: Supplementary file 4 — Additional file 4. (A) Association between total positive end-expiratory pressure (PEEP) and intensive care unit (ICU) mortality. (B) Association between plateau pressure and intensive care unit (ICU) mortality. [file 13054_2021_3667_MOESM4_ESM.pdf]

ADDITIONAL FILE 4

Title: Static compliance and driving pressure are associated with ICU mortality in intubated COVID-19 ARDS.

Authors: Annalisa Boscolo[1*] MD, Nicolò Sella[2*] MD, Giulia Lorenzoni[3] PhD, Tommaso Pettenuzzo[1] MD, Laura Pasin[1] MD, Chiara Pretto[2] MD, Martina Tocco[2] MD, Enrico Tamburini[2] MD, Alessandro De Cassai[1] MD, Paolo Rosi[4] MD, Enrico Polati[5] MD, Katia Donadello[5] MD, Leonardo Gottin[5] MD, Silvia De Rosa[6] MD, Fabio Baratto[7] MD, Fabio Toffoletto[8] MD, V. Marco Ranieri[9] MD, Dario Gregori[3] PhD, Paolo Navalesi[1,2] MD, FERS, for the COVID-19 VENETO ICU Network°.

**These authors equally contributed to this work.*

°Listed in the Acknowledgment section.

ADDITIONAL FILE 4.

A) Association between total positive end-expiratory pressure (PEEP) and intensive care unit (ICU) mortality.

Solid lines indicate the log-odds of ICU mortality, while grey areas 95% confidence interval. The p-value for non-linearity was 0.22, therefore non-linearity was not implemented in the model.

The odds ratio is presented for clinically relevant ranges of values, according to literature data [1].

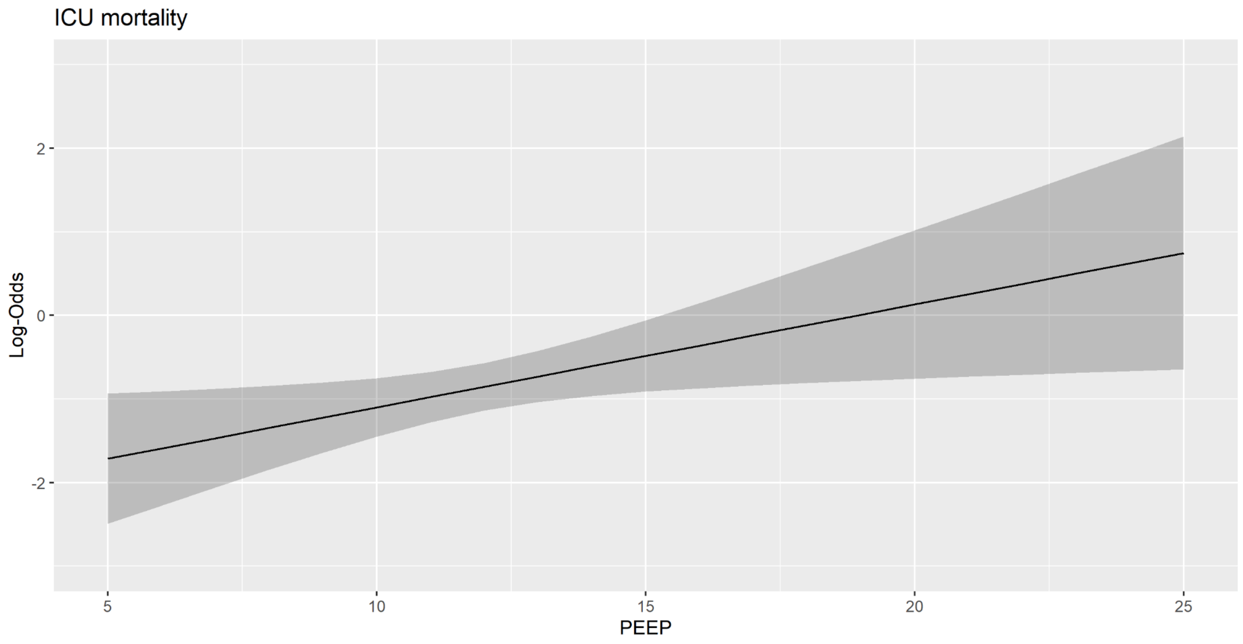

(cmH_2O)

**PEEP range** *(from - to)*	**Odds Ratio**	**95% Confidence Interval**
6-10 cmH_2O	1.64	1.08 - 2.48
10-16 cmH_2O	1.85	1.10 - 3.12
16-25 cmH_2O	3.03	1.18 - 7.75

B) Association between plateau pressure and intensive care unit (ICU) mortality.

Solid lines indicate the log-odds of ICU mortality, while grey areas 95% confidence interval. The p-value for non-linearity was 0.22, therefore non-linearity was not implemented in the model.

The odds ratio is presented for clinically relevant ranges of values, according to literature data [2].

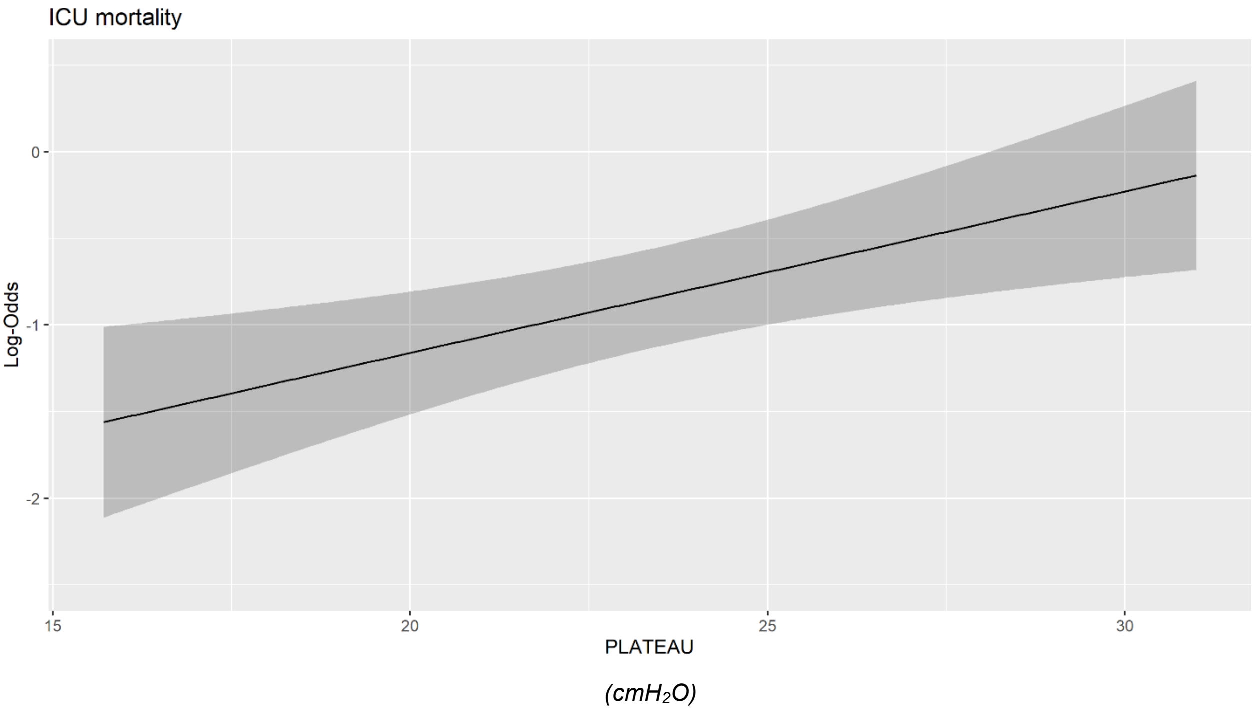

**Plateau pressure range** *(from - to)*	**Odds Ratio**	**95% Confidence Interval**
20-26 cmH_2O	1.75	1.21 – 2.52
> 26 cmH_2O	4.88	1.73 – 13.80

References

1. Brower RG, Lanken PN, MacIntyre N, Matthay MA, Morris A, Ancukiewicz M, et al. Higher versus lower positive end-expiratory pressures in patients with the acute respiratory distress syndrome. N Engl J Med. 2004; 351(4):327-36
2. Terragni PP, Filippini C, Slutsky AS, Birocco A, Tenaglia T, Grasso S, et al. Accuracy of plateau pressure and stress index to identify injurious ventilation in patients with acute respiratory distress syndrome. Anesthesiology. 2013 Oct;119(4):880-9
